# A Wicked Theft

**Published:** 1876-01

**Authors:** 


### A WICKED THEFT.

Stealing is a very bad practice; but some larcenies are worse than others. One of the monstrous sort just comes to our knowledge. In the number of *Hall's Journal of Health* for April, 1875, we published an article entitled "ELECTRICITY AND LIFE," which embodied the results of a prolonged and careful examination, on our part, of the electrical bands and other appliances called "THE VOLTAIC ARMADILLO," invented and manufactured by Mr. E. J. Seibert, now of No. 818 Broadway, New York. A pamphlet has been sent to us, seemingly published by "The Volta Belt Company," of Cincinnati, in which our article appears in distorted form, and is made to do duty for the concern which has thus wrested it from its intended purpose. Now, we know nothing about this "Volta Belt Company," or its belts; but we are inclined to look with suspicion upon the products of any house which is compelled to sustain itself by tricks of this nature. We do not complain that by distorting our language, and attaching the name of our *Journal* to the article, they compel us to father some very bad grammar. Our complaint is that by omitting words essential to a full comprehension of our meaning, they have taken from the "Armadillo" of Mr. Seibert, which we have proven to be valuable, the credit for excellence which we had so cheerfully given it, and have managed to make us appear to sustain their own appliances. This we look upon as dishonesty, and that of a very wicked type. These devices of Mr. Seibert are used and prescribed by a large number of physicians, and are relied upon by many citizens in every form of nervous disease. We know the opinions of several leading members of the profession, and are glad to publish a note from one of these:—

New York, July 13, 1875.

*Dear Sir:* I have prescribed the Voltaic Armadillo in a number of cases of *weakness of the back*, rheumatism, and several other forms of nervous affections, and the patients have expressed to me their entire satisfaction with them. For *weak back* in females it has been especially lauded by those for whom I prescribed it.

I am free to state there are many cases of a rheumatic and neuralgic character, for which these Bands and Soles offer a safe and almost certain help. To one of those stubborn cases of nervous headache which had gone the rounds of doctors and patent medicines, to little purpose, I *know* the Head-Band has given a comfort and ease the patient had actually despaired of again finding on earth. I cheerfully make these statements for the benefit of the profession, recommending a trial of the Armadillo in all suitable cases.

Julius Von Meyer, M.D.,
115 *West Twenty-Second Street.*

Rev. Dr. Tyng gives the following voluntary testimony:—

E. J. Seibert, 818 Broadway, corner Twelfth Street.

*My Dear Sir:* I have given a full trial to your Voltaic Armadillo, in separate applications, and cheerfully express to you my satisfaction with the personal advantage and benefit which I have received.

Stephen H. Tyng,
St. George's Rectory, 209 East Sixteenth Street.

New York, November 30, 1875.

Testimony of like character comes to us from Dr. Bahnson, of Salem, North Carolina; Dr. Sumner, of Mansfield Centre, Conn.; Dr. Greenleaf, of Owego, N. Y.; Dr. Sturdivant, of Millville, N. J.; Dr. Trueworthy, of Munice, Ind.; Dr. Bevier, of Owasco, N. Y.; Dr. Mallory, of Sherman, Conn.; Dr. Teackle, of Baltimore; Dr. Hackly, of 47 West Thirty-First Street, New York; and many other members of the medical fraternity. Of non-professional men and women there are multitudes who bear cheerful and earnest testimony to the benefits received from the use of these electrical devices. To publish the names of even a hundredth part of the vast army of intelligent sufferers who have gained comfort from these instruments, would occupy far too much of our space. We can only advise those of our readers who suffer from sleeplessness, spinal disturbance, nervous headache, or those more violent diseases involving the nerves, known as neuralgia and rheumatism, to write to Mr. Seibert for pamphlets descriptive of his valuable appliances.

